# Promoting recreational opportunities and experiences of students with disabilities, at a University, in Limpopo Province, South Africa

**DOI:** 10.3389/fspor.2025.1590372

**Published:** 2025-08-08

**Authors:** Anzani Mululuma, Ndiafhi Percy Mugwedi, Makhaya Malema

**Affiliations:** ^1^ Department of Sport, Recreation & Exercise Science, University of the Western Cape, Cape Town, Western Cape, South Africa; ^2^ Department of Biokinetics, Recreation & Sport Science, University of Venda, Thohoyandou, Limpopo, South Africa; ^3^ Department of Student Affairs: Sport and Recreation Unit, University of Venda, Thohoyandou, Limpopo, South Africa

**Keywords:** students, disabilities, recreational opportunities, recreational experiences, university

## Abstract

**Introduction:**

The Limpopo Province is one of the nine provinces in South Africa and has only two universities that serve students with disabilities coming from historically disadvantaged communities. The two universities within the Limpopo Province are categorically classified as Historical Disadvantaged Institutions of higher learning. Therefore, students with disabilities deserve the right to recreational opportunities and experiences while continuing with their tertiary education. A literature review confirmed an existing gap that relates to promoting recreational opportunities and experiences of students with disabilities in universities. The objective of the research was to gain a deeper insight into how recreational opportunities and experiences can be enhanced for students with disabilities at a university in Limpopo Province.

**Methods:**

The study adopted a qualitative research approach using an exploratory design wherein seventeen students with disabilities were purposively selected and consented to participate in the study. The study used semi-structured interviews to collect data.

**Results and Discussion:**

The study findings revealed three main themes, including recreation participation, benefits of active recreation, and more opportunities for participation. This indicates that students with disabilities do not fully participate in recreational activities due to limited accessible recreational activities, conducive facilities, and other societal challenges. The conclusion drawn is that recreational opportunities and experiences of students with disabilities are not adequately promoted in the University. This recommends the need for more exploratory mechanisms to address issues facing students with disabilities holistically.

## Introduction

The South African government developed, adopted, and implemented policies that protect all citizens, including people with disabilities. Chapter 2 of the South African Constitution (Act 108 of 1996) stipulates that all people, including those with disabilities, have the right to participate in recreational activities of their choice (1, 2). The Gauteng Provincial Government also developed, adopted and implemented disability policy to improve the lives of people with disabilities through recreational activity participation, which is in line with the Sustainable Development Goal number 10: Reduced Inequalities in order to promote full societal integration (3). This policy advocates for full participation in activities of daily living for people with disabilities and encourages a national intervention that will ensure that people with disabilities are part of mainstream society, having equal opportunities, living independently, and having an education, employment, and social integration. Furthermore, the provision of opportunities such as mass participation for meaningful experiences is a mechanism for students with disabilities for rehabilitation processes (4).

There is a need to develop recreational opportunities for students with disabilities throughout their university lives. Caldwell & Baldwin and Huesman et al. found that recreation programmes appeal to large segments of students with disabilities at tertiary institutions and offer service providers within campus recreation a way to positively influence students' pro-social behaviour such as peer interaction skills, teamwork, social acceptance and behavioural conduct (5, 6). Giles-Corti and Donavan conducted similar study and found that provision of equitable and efficient recreational opportunities has been reported to be low, thus it favours only the high socio-economic class (7). It remains unclear to assess whether opportunities such as participation pattern among students with disabilities in higher learning institutions may also be characterised by the imbalances of low participation (8).

Russell also indicated that recreation opportunities have the potential to make a significant contribution to the well-being of students with disabilities and building liveable campus community, not forgetting improving students' academic life (9). These mean that the absence of recreational opportunities may contribute to sedentariness and unhealthy campus community within the tertiary institution which is supported in (10). This includes providing relevant and accessible facilities, equipment and recreational activities. Recreational activities are defined as any indoor or outdoor physical activity, sport, team sport, or game, whether organised or unorganised, undertaken for the purpose of exercise, relaxation, diversion, education, or pleasure, including practice or instruction in any such activity (11–13).

Morres et al. assert that it is significant to develop effective strategies to promote recreational programmes that accommodate students with disabilities and students without disabilities (14). This intervention will ensure that students with disabilities improve their confidence and participate effectively in activities designed for personal development (15). It is critical for institutions of higher learning to collaborate with non-profit organisations to increase resources and expertise in offering recreational activities for students with disabilities (16). The universities have the responsibility to equip their staff with skills and knowledge on how to work with students with disabilities, including on how to communicate with them and how to manage their behaviour (17).

Literature review indicate that there are insufficient recreational opportunities for students with disabilities (12), and limited research on the key factors that are necessary to consider when planning and implementing recreational opportunities for students with disabilities (18, 19). Factors to consider include availability of facilities such as golf or tennis courses; accessibility, inclusive policies and environmental factors, such as weather patterns (20). There is also a limited understanding of the significance of recreational opportunities for students with disabilities within higher education institutions. The South African higher learning institutions lack inclusive recreational opportunities for students with disabilities. Students with disabilities may lack opportunities to develop life skills and to have developmental experiences concerning social interaction, choice, and personal growth (21).

Sheids and Synnot argue that many educational establishments do not provide students with disabilities with sufficient support to engage in recreational activities designed to promote a healthy lifestyle for them (15). To guarantee conducive environments, strategies and plans to accommodate students with disabilities are necessary. The Universities worldwide adopts an inclusive approach to accommodate, include and supports students with different disabilities. These include students with visual, hearing, physical and speech impairments, chronic illnesses (for example, epilepsy), back injuries, carpal tunnel syndrome, bipolar disorder, and severe anxiety or depression. In ensuring that these strategies are realised in the form of recreational experiences, a large number of health benefits were identified by Caldwell and Baldwin in recreational participation and has been linked to the prevention of lifestyles related diseases and absence of sickness and diseases such as hypertension, obesity, overweight and heart attack, improved physical fitness and cardiovascular system (5, 20, 22–24).

## Methods

### Design

The study adopted a qualitative research approach using an exploratory design. The primary objective of exploratory research is to gain insights and gather preliminary information that can help the researcher better define the research problem and develop research questions for further investigation (25). Creswell indicates that exploratory design helps the researcher to identify patterns, themes and relationships (25). As such, the researcher will be able to identify areas for further research. The present study was conducted at the University of Venda, Limpopo Province, South Africa. The University of Venda (Univen) is a rural-based university established in 1982 during the apartheid era in the Thohoyandou area in the Northern Province of Limpopo. Generally, within the South African contexts, the Univen is classified as a Historically Disadvantaged Institution (HDI).

### Sampling process and procedure

The current study used purposive sampling to recruit 17 students with Physical disabilities registered at Univen during the 2024 academic year. Table 1 illustrates the demographic information of the participants of this study. The purposive sampling technique is used when selecting a sample of participants based on the experience they have regarding a particular phenomenon. This method was chosen due to the practical constraints of reaching senior-year students, who were often engaged in clinical rotations. Consequently, the sample was drawn from available students within the premises. The study did not explicitly mention accommodation status as a parameter. This also helps researchers identify the study population in a consistent, reliable and objective manner. The researcher collected data and reached saturation during the fifteenth interview; additional two interviews were done post data saturation to maintain rigor.

**Table 1:** Demographic characteristics of participants.

Participant	Gender	Age	Level of study	Accommodation status
SwD1	Male	22	First year	On-campus
SwD2	Female	25	First year	Private accommodation
SwD3	Female	21	Second year	On-campus
SwD4	Male	20	Fourth year	Off-campus
SwD5	Male	20	Fourth year	Private accommodation
SwD6	Male	26	Third year	Private accommodation
SwD7	Female	26	Third year	Private accommodation
SwD8	Female	19	Second year	Off-campus
SwD9	Female	19	First year	On-Campus
SwD10	Female	21	Third year	On-campus
SwD11	Female	22	Second year	Private accommodation
SwD12	Male	22	Fourth year	On-campus
SwD13	Male	25	Fourth year	Off-campus
SwD14	Female	21	Second year	On-campus
SwD15	Female	18	Third year	Off-campus
SwD16	Male	25	First year	On-campus
SwD17	Female	24	Fourth year	On-campus

### Participants information

The present study adopts an inclusive approach in terms of participants represented in the current study. Auricchio and Leyva defines inclusion and exclusion criteria as the characteristics that prospective participants must have if they are to be included in a study (26). The researcher selected only participants who gave their consent to participate in the study. This study included students with various disabilities registered for the 2024 academic year, aged between 18 and 35 years. Further details of the participants are reported under Table 1 below.

### Data collection process

The researcher consulted with the Disability Unit Manager and school registrar for permission to approach students with disabilities on the premises. After receiving permission, the researcher approached and recruited students with disabilities, requesting their consent for participation. The researcher used a semi-structured interview guide using open-ended questions to conduct interviews. According to Kallio et al. when using an interview guide for a semi-structured interview, the interviewer should not strictly follow a formalised list of questions (27). According to Richardson an open-ended question is one that allows the respondent to express himself or herself freely on a given subject (28). Interviews were conducted in English, which is the official language of instruction at Univen. Each interview lasted approximately 50 min. Interview questions covered topics on current recreational opportunities, available equipment and resources, and involvement in the programs on campus.

### Data analysis

**Figure 1:**
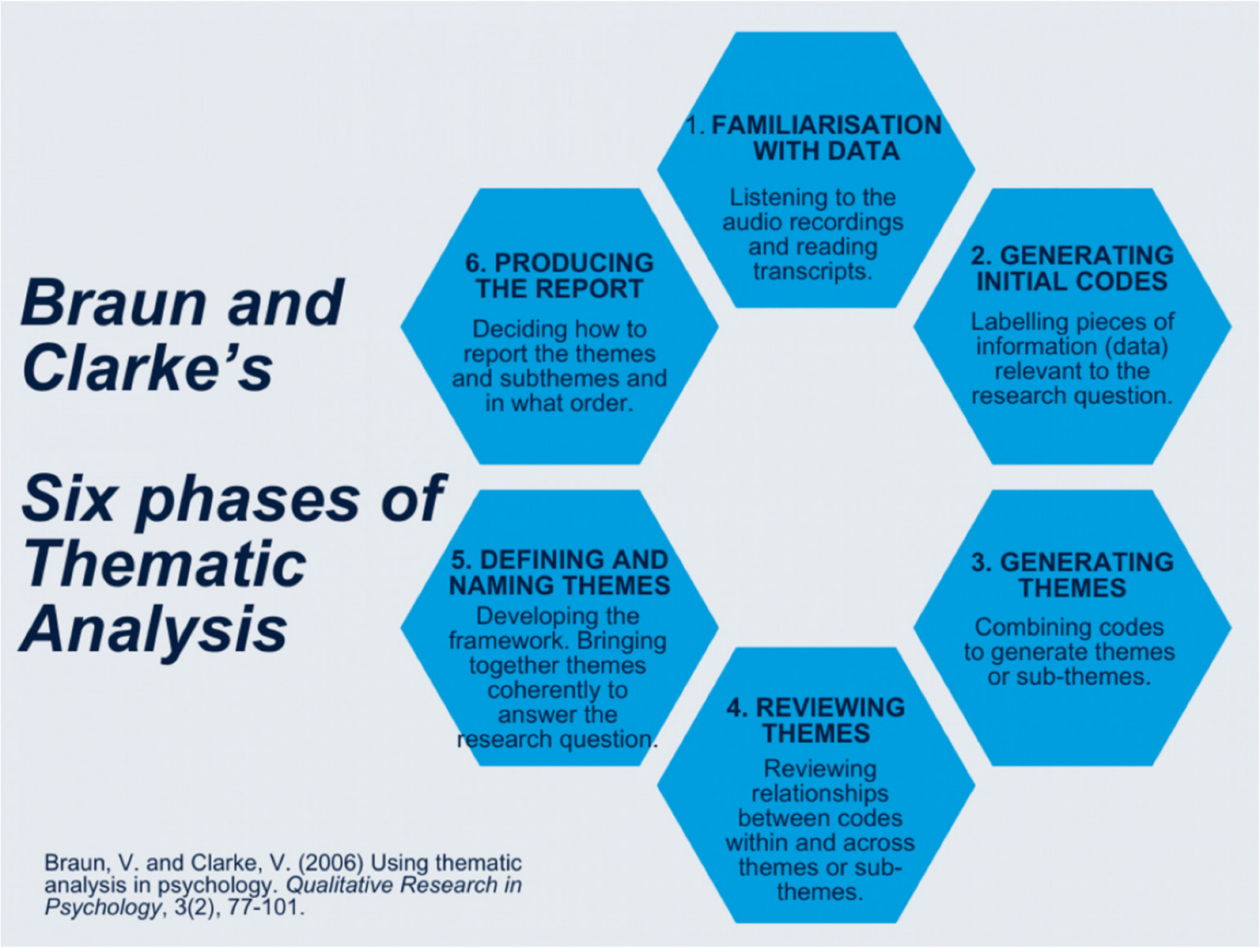
Thematic analysis diagram.

For the data analysis framework, this study adopted Braun and Clarke's six-step framework (29). This framework provided a systematic, sequential and logical structure for data analysis (see Figure 1). First, the recorded interviews were transcribed ad verbatim. Secondly, the researcher became familiar with the data by reading and re-reading through the raw transcript texts and taking down initial notes, general thoughts and ideas (29). The third step involved data coding. Using Atlas.ti, two coders independently assigned codes to segments of data. The coders then discussed the codes and refined them for consistency (29). The patterns that emerged from recurrences and common meanings from the codes were discussed to address areas of agreement or disagreement (29). The fourth step involved identifying patterns among the codes to come up with themes. The emerging key themes were named and identified. The fifth step involved examining how the themes and descriptions can be represented as a qualitative narrative (29). In the sixth and final step, the key implications of the themes were discussed and interpreted in relation to the literature to draw key conclusions (29).

### Ethics

Ethical considerations were important in this study because they promoted authenticity, originality, and true knowledge by avoiding error. They promoted the values of collaborative work and ensured public accountability. In addition, ethics help to ensure quality and integrity along with moral and social values such as social responsibility, human rights, legal compliance, animal welfare, and public health and safety (30). As such, this study was submitted and presented before the Humanities and Social Science Research Committee (HSSREC) of the University of Western Cape for approval. Permission to conduct the study among students with disabilities within the University of Venda was obtained from the disability office unit. The participants were given information prior to the interviews so that they understood what the study was all about and what was expected of them. Informed consent forms were distributed to participants to sign before the interviews. The researcher informed participants that their participation is on a voluntary basis and that they are allowed to quit at any time should they feel uncomfortable. The researcher maintained the anonymity of the participants by not using their real names. Participants were assured that their interview records would be kept in lockable cabinets and would thereafter be destroyed when the complete research had been approved. Participants were assured that their information would not be shared with anyone without their permission. All the data collected during interviews was kept safe by the researcher and supervisors in an encrypted file that is password protected.

## Findings

The participants were students with disabilities. Seventeen participants were recruited and selected using the purposive sampling method. The responses from the participants shed light on their experiences and views of recreational opportunities. The results are presented in different themes and sub-themes.

### Theme one: recreational activity experiences

In this theme, it was important to understand the participants perceived knowledge about what recreational opportunities are, their experiences of programs and participation levels in such programs. Participants, generally understood the concept of recreational opportunities, however, this was limited to their exposure, in terms of activity participation, and what they could experience from the TV and other platforms. Under this theme, participants expressed their experiences and reflects about their encounters within various recreational programs at their campus. This theme is supported by three subthemes in Table 2 and representative quotes from participants below.

**Table 2:** Subtheme findings and sample quote for theme one.

Subtheme	Representational quote
Meaning of recreational activity	“*I think for me recreation is just talking about activities that you can do in your spare time or in your free time. Many activities that you can join are available on my free time those are types of activities that i can do is either as a hobby or as an exercise those are recreational activities*” (SD 1). “*Is when one partakes in activities in his free time or when one is not working*” (SD 4). “*Recreation according to me is when you go and participate in any activity when you are free it can a soccer, netball or basketball*” (SD 7). “*When we talk about recreational activities we are referring to activities done during leisure, so basically when we participate in these activities we do it for enjoyment and to socialise with other students*” (SD 13).
Recreational activity preferences	“*I am not participating in recreational activities as yet, but I would love to join to join basketball*” (SD 14). “*I participate in goal ball but I do not go practice every day, I feel like it is not helpful to me because when there is tournament they do not include me. Therefore, I feel discriminated*” (SD 6).
Given the opportunity	“*I would like to participate in chess to help me with cognitive skills, boost selfesteem and stress relief.*” (SD 1). “*I love basketball and wheelchair tennis, but I can only participate if they give us wheelchairs for basketball because I don't want to hurt myself. I tried to be used mine and I nearly fell during participation trying to move part to catch the ball*” (SD 12). “*I would like to participate in volleyball, I believe that I can be a great player because I have passion for it*” (SD 9). “*I love badminton, I would love to be part of the team should I be given the opportunity*” (SD 6).

### Subtheme: meaning of recreational activities

From the findings above under this subtheme, it can be deduced that participants have a similar understand of the concept of recreation within their context. This shows the universal understanding of the concept and how it links up with global trends about the association of recreation from all persons. Within the context of the present study, it is important to understand the meaning attached to the concept of recreational activities, to further understand the various domains of how these looks like in their daily lives as persons with disabilities. The participants linked the concept of recreational activities to free time and programs that promotes free expression and socialization, which is in line with the literature.

### Subtheme: recreational preference

In terms of the preferred recreational activities, participants under this subtheme reported some active recreational activities. These activities are not extra-ordinary, rather the same activities that can be accessed by all individuals. The recreational activities for people with disabilities can be modified, adapted to accommodate their levels of participation and support required. Notwithstanding the challenges that is associated with inclusive sport, such as specialized equipment. The concept of inclusive sport becomes applicable and promotes greater participation levels for people with disabilities.

### Subtheme: given the opportunity

In this subtheme, participants shared that there are some recreational activities programs that they would want to be part of. The recreational activities programs reported under this subtheme exist at the university campus, however, it appears that the participants are not being accommodated to participate in them. Similarly, the recreational activity programs reported under these subthemes are similar to programs that students without disabilities participate in. It is unfortunate that students with disabilities are marginalized within the campus space, and are not accommodated and supported regarding their recreational needs and preferences.

### Theme two: benefits of recreational programs

Under this theme, participants offer insights about the significance of being part of recreational activity programs and opportunities that exist on their campus. Given the historical context of Univen, it could be fair to say the recreational programs within the campus, could be linked to the cultural influences of traditional games and activities. Due to the unique characteristics of the participants in this study, it is feasible that participants could experience recreational programs differently than their counterparts. It is undeniable that people with disabilities can participate in recreational activities if they are afforded similar opportunities and access to such programs. Table 3 below illustrate the subthemes and sample quotation to support the findings for theme two.

**Table 3:** Subthemes and sample quote for theme two.

Subtheme	Sample quotation
Personal benefits	*“As students we go through a lot, especial in academics. So, when you go out and participate in recreational activities with another student. Participating in recreational activities reduce stress and boosts self –Esteem”* (SD 4). *“Recreational activities improve blood circulation, cardiovascular fitness, enhances muscles and improve flexibility. It can also help to burn Calories, which is an effective way to prevent diseases such as Diabetes and others”* (SD 5). *“When I participate in recreational activities I gain new skills like problem solving skills. I learn how to use my skills to win the game”* (SD 12)*.*
Societal benefits	*“When we participate in recreational activities we interact we formulate friendship. I can say that recreational opportunities play a huge role in reducing discrimination and stigma, we feel the sense”* (SD 2). *“Participating in recreational activities build my character… it has taught me how to interact with others and make friends. Recreational participation helps us to meet new people and be able to share ideas. We feel good to be around other students”* (SD 13). *“Recreational participation enhances social cohesion and foster social change. We get to learn how to socialize with other students*” (SD 16).

### Subtheme: personal benefits

Recreational participation helps to stay in shape, teach how to organise time and build relationships with other people. The participants indicated that recreational participation plays a huge role in their well-being, resilience and social support as students with disabilities. Participants' responses show that some of them are aware of the benefits they get when they engage in recreational activities. This indicates that their recreational activities hold no boundary in terms of benefits.

### Subtheme: societal benefits

Participants regard recreational activities as a powerful resource for students with disabilities to connect with their peers. The responses from the participants reveal that recreational participation plays a huge role in reducing discrimination against students with disabilities. Recreational participation has demonstrated social benefits, which promote quality of life. The participants indicated that when they participate in recreational activities with other students, they gain confidence and build friendships. This shows that more could be done to ensure that all students with disabilities enjoy the benefits of sharing ideas with other students during participation.

## Discussion

The aim of the study was to explore a better understanding on how recreational opportunities and experiences can be promoted for students with disabilities at a University in Limpopo Province. Two main themes: Recreational activity experiences and Benefits of recreational programs emerged from the study, along with five subthemes: Meaning of recreational activities; Recreational preference; Given the opportunity; Personal benefits and Societal benefits were reported. The findings of this study indicate that students with disabilities are not aware of some of the activities offered in their institutions, and the recreational facilities are not accommodating enough to allow them to go and watch when other students participate.

The participants revealed that societal challenges, limited recreational programmes, inaccessible facilities contribute towards non-participation, which affects them academically and mentally. Students with disabilities shared that when they participate in recreational activities, they feel discriminated against and isolated as those who do not have disabilities play against each other. They further indicated that when it is time for them to play, no one is watching them or to give them courage. This indicates a need for inclusive participation and for mixing participants during activities, ensuring that they feel a sense of belonging and appreciation. This will improve their results and mental well-being.

Inaccessibility to recreational facilities has limited the opportunities of students with disabilities to engage in social and recreational activities. The study conducted by Ngobeni reports that most people using wheelchairs are unable to gain access to recreational facilities due to accessibility barriers (31). The present study highlights the need for recreational facilities to be equally accessible to all students. Disability-specific programmes and awareness campaigns should be done more often to empower students with disabilities and to eradicate the misconception that other students have towards them. Furthermore, the facilities should be equitably distributed, carefully planned and well managed to serve all students with disabilities. The development of accessible recreational facilities for students with disabilities to quality physical activity and recreational opportunities should be addressed on an equal basis with students without disabilities (32). It is therefore crucial to provide accessible and inclusive activities that cater to the diverse needs of students with disabilities, ensuring that they can fully participate and benefit from these opportunities.

Lack of awareness from people without disabilities on how to include people with disabilities in sport, lack of opportunities for training and competition, accessible facilities and limited resources, and negative social attitudes are also reasons for non-participation in recreation activities, causing social isolation and impact on emotional and physical wellbeing (33). Therefore, perceptions can often change when students are mixed, engaging people who are differently abled in an adaptive sport setting, for example in basketball. Recreational activities are encouraged for students with disabilities to promote mental and physical well-being. Inclusive recreational activities can also have a positive influence on participation engagement (34). By addressing these limitations, we can ensure equal access to recreational opportunities and promote a more inclusive and equitable society for students with disabilities. Regular participation can improve concentration, motivation and overall academic performance to students with disabilities.

The findings in the present study revealed that recreational programmes can contribute to improving the lifestyle of students with disabilities. They can increase competency in gross motor skills, improve social skills and encourage an active lifestyle. Recreational participation provides significant benefits for every individual in all developmental stages of life. The findings from the current study reiterate the benefits reported from the available literature. Herbison et al. argue that regular participation in recreational activities would promote positive advancements in students with special needs and improve their performance such as academics and boost their confidence (32). The benefits of recreational activities are universal for all human beings.

Ballas et al. indicate that the international efforts to promote the social and emotional wellbeing of children with disabilities through participation in recreational activities began with the first competitive event for people with disabilities in 1948 (33). This indicates that students with disabilities should be provided with competitive events for them to be empowered and to be part of the community. This will help to maintain normal adaptive behaviours and fatigue in students after a long day of attending classes. The participants indicated that recreational activities foster independence, coping abilities, teamwork and socialisation.

Participants in the current study revealed that regular participation leads to improved levels of well-being and physical health. This shows that recreational participation is an effective way of reducing chronic diseases and low self-esteem. However, some of the participants revealed that despite the benefits acquired from recreational activities, students with disabilities are not taken seriously; they are not getting the same support and programmes as other students without disabilities. Students with disabilities are experiencing inequalities in recreational opportunities, which has a negative impact on their health and well-being. Therefore, there is a need for a comprehensive change to address these difficulties, exclusion and discrimination against students with disabilities. Recreation activities are essential for promoting social well-being, mental, personal development and physical well-being. Through recreational participation, students can develop essential life skills, self-esteem and build confidence. They learn how to manage stress, anxiety and depression.

Additionally, the findings from the present study revealed that when participants interact with each other, they develop problem-solving skills and teamwork. They indicated that recreational participation promotes socialisation and inclusion. Howie et al. emphasise that by promoting inclusion, socialisation and empathy, we can play a huge role in improving health and wellness (35). It fosters social interactions, building relationships and creating a sense of community. These aspects of social interaction can improve the mental health and well-being of a community. Amusa et al. indicates that recreational participation can transcend social and cultural barriers (36). It promotes inclusivity and the well-being of students with disabilities. Disability sports and recreation activities are increasingly becoming a powerful tool for positive change in our society, and there are options available.

Jones indicate that recreation activities create bonds with others and help individuals feel part of the community, they teach individuals how to navigate social situations effectively, communicate and learn to solve problems with others (37). By challenging stigma and discrimination, we can create a more inclusive and equitable society that values diverse participation and promotes overall well-being. Exclusion from recreational activities can limit socialisation and skill-building opportunities among students with disabilities (38, 39). Therefore, by addressing these societal challenges, we can create a more inclusive environment that fosters academic success, personal growth and social inclusion for students with disabilities. Socialisation and recreational participation are the key components of our daily life, therefore, students living with disabilities should not be left behind, and they should also enjoy the benefits like other students living without disabilities. Weybright et al. indicate that life without any form of recreational activities can become dull and cause a negative impact on our overall health and well-being (40). Keeping students physically active and mentally stimulated is beneficial to their academic performance and fosters positivity.

## Recommendations

The following recommendations were made based on the findings obtained from the participants: the university should implement unified sports programmes, where athletes with and without disabilities compete together. This will provide opportunities for students with disabilities to participate alongside their non-disabled peers, promoting social inclusion and acceptance. Provide adaptive sports equipment, such as prosthetic limbs or wheelchairs, to facilitate participation. Adapted sports equipment promotes inclusive participation by providing the necessary tools for students with disabilities to engage in sports and physical activities, fostering a sense of belonging and promoting equal opportunities for all students. The university, together with the Disability Unit within the university, should create awareness and educate students about the benefits of recreational participation. There should be a more inclusive and supportive environment for students with disabilities to participate in sports and reach their full potential. Tertiary institutions should promote participation and provide resources such as assistive devices and accessible recreational facilities for students with disabilities.

## Data Availability

Data presented on this manuscript is available on reasonable request to the corresponding author.
